# Regulation of Phytosiderophore Release and Antioxidant Defense in Roots Driven by Shoot-Based Auxin Signaling Confers Tolerance to Excess Iron in Wheat

**DOI:** 10.3389/fpls.2016.01684

**Published:** 2016-11-10

**Authors:** Ahmad H. Kabir, Most A. Khatun, Mohammad M. Hossain, Syed A. Haider, Mohammad F. Alam, Nishit K. Paul

**Affiliations:** Plant and Crop Physiology Laboratory, Department of Botany, University of RajshahiRajshahi, Bangladesh

**Keywords:** phytosiderophore release, Fe-toxicity, wheat, auxin signaling, reciprocal grafting, 2-DMA

## Abstract

Iron (Fe) is essential but harmful for plants at toxic level. However, how wheat plants tolerate excess Fe remains vague. This study aims at elucidating the mechanisms underlying tolerance to excess Fe in wheat. Higher Fe concentration caused morpho-physiological retardation in BR 26 (sensitive) but not in BR 27 (tolerant). Phytosiderophore and 2-deoxymugineic acid showed no changes in BR 27 but significantly increased in BR 26 due to excess Fe. Further, expression of *TaSAMS. TaDMAS1*, and *TaYSL15* significantly downregulated in BR 27 roots, while these were upregulated in BR 26 under excess Fe. It confirms that inhibition of phytosiderophore directs less Fe accumulation in BR 27. However, phytochelatin and expression of *TaPCS1* and *TaMT1* showed no significant induction in response to excess Fe. Furthermore, excess Fe showed increased catalase, peroxidase, and glutathione reductase activities along with glutathione, cysteine, and proline accumulation in roots in BR 27. Interestingly, BR 27 self-grafts and plants having BR 26 rootstock attached to BR 27 scion had no Fe-toxicity induced adverse effect on morphology but showed BR 27 type expressions, confirming that shoot-derived signal triggering Fe-toxicity tolerance in roots. Finally, auxin inhibitor applied with higher Fe concentration caused a significant decline in morpho-physiological parameters along with increased *TaSAMS* and *TaDMAS1* expression in roots of BR 27, revealing the involvement of auxin signaling in response to excess Fe. These findings propose that tolerance to excess Fe in wheat is attributed to the regulation of phytosiderophore limiting Fe acquisition along with increased antioxidant defense in roots driven by shoot-derived auxin signaling.

## Introduction

Fe (Fe) is essential for plant growth and development, but its presence in soil at toxic level causes reduced the agricultural productivity of many crops (Dobermann and Fairhurst, 2000; Finatto et al., 2015). Excess Fe accumulation mainly occurs due to high active Fe, soil acidity, reduced oxides, and waterlogged conditions (Connolly and Guerinot, 2002). In addition, excess Fe is closely linked with aluminum availability in the ground irrespective of organic matter and texture (Garrity et al., 1986). Growth factors, such as age, organic matter, and hydrogen sulfide accumulation may also generate Fe-toxicity in plants (Sahrawat, 2004). Furthermore, low soil pH and flooded soil conditions (rainy season) lead to the reduction of Fe^3+^ to Fe^2+^ causing excess Fe (Fageria et al., 2011). Importantly, Fe-toxicity is a serious agricultural problem, particularly when plants are grown in acidic soils.

Among the two oxidation states of Fe, Fe^2+^ is more constant in soil, which is readily absorbed by plant roots and is involved in the metabolic imbalance and oxidative tissue damage in plants (Li et al., 2015). Leaf bronzing, poor root development, and suppression of tissue cation levels are common symptoms of Fe-toxicity in plants (Becker and Asch, 2005; Aung, 2006; Çelik et al., 2010). Further, Fe-toxicity may cause decreased mineral uptake, protein degradation, and enzymatic activities along with the induction of reactive oxygen species (ROS) in plants (Sandalio et al., 2001).

Plants have developed few strategies to limit free Fe accumulation in tissues. These include exclusion of Fe in roots, oxidation, and formation of Fe plaques in the rhizosphere, avoidance of Fe via internal distribution into vacuole, inclusion in ferritin proteins, and antioxidant defense (Audebert and Sahrawat, 2000; Fang et al., 2001; Pich et al., 2001; Wu et al., 2014). Hence, maintenance of tissue Fe and related cations is often associated with Fe-toxicity tolerance in plants. Graminaceous monocots or Strategy II plants release Fe-chelating substances, deoxymugineic acid (DMA) of the family phytosiderophores (PS) into the rhizosphere to solubilize inorganic Fe followed by Fe^3+^–DMA association before taken up the Fe in root plasma membrane (Nakanishi et al., 2000; Kim and Guerinot, 2007; Ishimaru et al., 2010). However, metal chelation is not specific for Fe^3+^, and it may mobilize a broad range of metals, such as Zn, Cu, Mn, Ni, Cd, etc. (Shenker et al., 2001). Further, wheat plants only exude 2-DMA in relatively small amount (Kim and Guerinot, 2007). Bashir et al. (2006) isolated *TaDMAS1* gene responsible for DMA synthesis from wheat involved in Fe homeostasis. In rice, upregulation of *OsDMAS1* is involved in Fe homeostasis and long-distance transport (Bashir and Nishizawa, 2006). Once the metal is chelated in the rhizosphere, PS uptake in root cells is mediated by membrane proteins of the yellow stripe 1/yellow stripe like (YS1/YSL) family having a high affinity for PS-chelated Fe^3+^ (Curie et al., 2001; Schaaf et al., 2004). The genes related to DMA regulation have been studied in a few graminaceous plants (i.e., rice, maize, etc.); however, yet to be studied in response to higher Fe concentration in wheat. *OsYSL15* has been recently reported to be involved in the uptake and phloem transport of Fe as the dominant Fe-DMA transporter in rice (Inoue et al., 2009). Another transporter gene, *OsYSL2* is responsible for the phloem transport and translocation of Fe into the rice grain (Ishimaru et al., 2010). As a grass species, wheat would also be expected to have YSLs that transport PS. Plants also produce low-molecular-weight chelators, such as phytochelatin (PC) and metallothionein (MT) to detoxify metals. PCs do have high affinity to bind with metal (mostly Cd) and ship the metal-PC complexes to root vacuole as their final destination (Cobbett, 2000; Song et al., 2014; Emamverdian et al., 2015). MTs also facilitate cellular sequestration and homeostasis of the metal ion in plants leading to safe storage (Memon et al., 2001; Kohler et al., 2004). Besides these, phenolic compounds are suggested to enhance Fe availability and are induced following Fe deficiency to solubilize insoluble Fe through its chelating and reducing ability (Dakora and Phillips, 2002; Jin et al., 2008). However, its role on Fe-toxicity tolerance in plants is not yet clear.

Plants possess complex antioxidant defense through antioxidant enzymes and metabolites for scavenging ROS generated due to abiotic stress (Dat et al., 2000; Kabir, 2016). Antioxidant enzymes, such as catalase (CAT), peroxidase (POD), glutathione reductase (GR), superoxide dismutase (SOD), and ascorbate peroxidase (APX) are generally induced under metal stress in plants (Cuypers et al., 2011; Kabir et al., 2015; Kabir, 2016). In addition, some endogenous metabolites (i.e., cysteine, glycine, alanine) play critical roles to alleviate the damage induced by metal stress (Sharma and Dietz, 2006; Kumar et al., 2014; Kabir, 2016). In particular, glutathione (GSH) is known to have a central role for nullifying metal stress in plants (Freeman et al., 2004; Kabir, 2016).

Mechanisms conferring tolerance to abiotic stress are often activated through signals transmitted from roots to shoot or vice-versa. Origin of these signals could either be in root or shoot before being forwarded to the source of stress to trigger biochemical processes (Kabir et al., 2013; Begum et al., 2016). Reciprocal grafting of pea genotypes previously showed that Fe deficiency signal is generated in the shoot before triggering the tolerance mechanisms in roots (Kabir et al., 2013). Again, As-tolerant mechanisms are driven by the signal originated in the roots of rice plants (Begum et al., 2016). To date, the grafting experiment in wheat underlying Fe-toxicity tolerance has never been reported before in the scientific literature. However, the origin and types of signal involved in stress tolerance are complex and species dependent. Hormonal networks control various aspects of growth, cell wall plasticity, and abiotic stress tolerance in plants (De Tullio et al., 2010; Tognetti et al., 2010; Kabir et al., 2013). Under stress, the hormonal signal often elicits changes specific protective mechanisms. Auxin is known to have association with abiotic stress tolerance in plants (Lequeux et al., 2010; Bacaicoa et al., 2011; Kabir et al., 2013). Recent studies demonstrated that systemic auxin signaling is associated with Fe-deficiency-induced growth inhibition and photosynthesis suppression in rice (Liu et al., 2015).

Fe-toxicity is a critical agronomic problem causing yield loss in wheat. However, our understanding of the mechanisms underlying Fe-toxicity tolerance in wheat remains unknown. Therefore, we investigated morphological features, Fe acquisition, and physiological parameters to confirm the differential genotypic variations in roots and shoots following Fe-toxicity in contrasting wheat genotypes (BR 27 and BR 26). To elucidate the mechanisms, several biochemical mechanisms, and their related genes were extensively studied. Moreover, antioxidant enzymes and plant metabolites were analyzed if their changes may trigger Fe-toxicity tolerance in wheat. We also sought to determine the origin and type of hormonal signal driving the Fe-toxicity tolerance by reciprocal grafting and hormonal inhibitors.

## Materials and Methods

### Plant Cultivation

Seeds of two wheat cultivars, BR 27 (rust resistance, pedigree: Waxwing^∗^2/Vivitsi, CIMMYT breeding line: Francolin #1) and BR 26 (heat and leaf blight resistant, pedigree: ICTAL123/3/RAWAL87//VEE/HD2285), with different tolerance to excess Fe, were used in this study, the former being tolerant and latter sensitive. Following surface sterilization of seeds with 75% ethanol and deionized water, seeds were germinated in the dark at room temperature. Afterward, uniform seedlings were transplanted to the solution culture (Hoagland and Arnon, 1950) containing the following nutrient concentrations (μM): KNO_3_ (16000), Ca(NO_3_)_2_.4H_2_O (6000), NH_4_H_2_PO_4_ (1000), MgSO_4_.7H_2_O (2000), KCl (50), H_3_BO_3_ (25), Fe-EDTA (25), MnSO_4_. 4H_2_O (2), ZnSO_4_ (2), Na_2_MoO_4_.2H_2_O (0.5), and CuSO_4_.5H_2_O (0.5). Plants were grown in 2 L of the aerated solution in a growth chamber under 10 h light and 14 h dark (550–560 μmol s^-1^ per μA). The pH was adjusted to 6.0 by using NaOH or HCl. Higher Fe concentration was imposed by adding 15 mM Fe-EDTA to the culture solution in the light of preliminary study (**Supplementary Figure S1**). All control and stressed plants were grown concurrently for 4 days after treatment was imposed and harvested at the same time.

### Measurement of Growth Parameters and Chlorophyll Concentration

Root length and shoot height of each plant were measured using a scale. Further, separated root tissue was washed with deionized water and blotted in tissue paper. Afterward, both root and leaf tissues were dried at 80°C for 2 days before measuring the dry weight in digital balance. For chlorophyll (a and b) determination, freshly harvested shoots were weighed and ground with 2 ml methanol using mortar and pestle. The homogenate was then centrifuged at 12000 × *g* for 10 min and the clear supernatant was placed in a centrifuge tube. The absorbance was read at 662 (chlorophyll a) and 646 (chlorophyll b) on a spectrophotometer (UV-1650PC, Shimadzu) and calculated as previously described (Lichtenthaler and Wellburn, 1985).

### Determination of Fe in Plant Tissues

Harvested tissues were washed with CaSO_4_ (1 mM) and deionized water before drying in an oven at 80°C for 3 days (Kabir et al., 2015). Once tissues were dried, 3 mL HNO_3_ and 1 mL of H_2_O_2_ (hydrogen peroxide) were mixed with samples and heated at 75°C for 10 min. The concentration of Fe was then analyzed by Flame Atomic Absorption Spectroscopy (AAS) outfitted with an ASC-6100 autosampler and air-acetylene atomization gas mixture system (Model No. AA-6800, Shimadzu). Standard solutions of Fe were separately prepared from their respective concentration of stock solutions (Shimadzu).

### Determination of PS Releases in Roots

PS release in roots was analyzed by determining the PS content in root washings. Briefly, wheat plants were removed from the hydroponic conditions 2 h after the onset of the light period and washed with deionized water for 1 min. Harvested roots were then submerged in 500 ml distilled water aerated by the hydroponic pump for 3 h. Afterward, collected exudates were passed through micropur (Roth, Germany) to prevent microbial degradation of PS (Valentinuzzi et al., 2015). After that, exudates were filtered through filter paper and concentrated to 20 ml at 50°C under vacuum. Finally, PS release was determined as whole plant release and per gram dry weight in roots following Fe-binding assay as previously described (Reichman and Parker, 2006).

### Estimation of Total Soluble Protein

Total soluble protein in both roots and shoots were measured using the calibration curve of different concentration of bovine serum albumin (BSA) as previously described (Guy et al., 1992). Briefly, roots and shoots were harvested and washed with deionized water before homogenization with a chilled mortar and pestle in a buffer containing ice-cold 50 mM Tris-HCl, pH 7.5; 2 mM EDTA and 0.04% (v/v) 2-mercaptoethanol. The homogenate was then centrifuged at 12000 × *g* for 10 min at room temperature, and clear supernatant (100 μl) was transferred to glass cuvette containing 1 ml Coomassie Brilliant Blue. Finally, the absorbance was read at 595 nm in a spectrophotometer, and the concentration of total soluble protein was calculated using the calibration curve of BSA.

### Measurement of Electrolyte Leakage

Electrolyte leakage (EL) of roots and shoots was measured by an electrical conductivity meter (Lutts et al., 1996). Briefly, harvested roots and shoots were washed with deionized water to remove surface contaminants and submerged in a vial containing 20 mL deionized water. The samples were then incubated at 25°C on a shaker (3000 g) for 2 h. Finally, the electrical conductivity of the solution was measured with the meter.

### Measurement of Total Phenol Content

Total phenol content in roots and shoots was analyzed by Folin–Ciocalteu’s method (Kogure et al., 2004). Root and shoot extracts were mixed with mixed with 250 mL of Folin–Ciocalteu’s phenol reagent and 20% Na_2_CO_3_ solution. The samples were then incubated at room temperature for 30 min before measuring the absorbance at 765 nm in a spectrophotometer. Finally, total phenol content was measured using the calibration curve of gallic acid.

### Analysis of Enzymatic Activities and H_2_O_2_ Production

Catalase, peroxidase, superoxide dismutase, and glutathione reductase enzymes were extracted in roots as previously described with slight modifications (Goud and Kachole, 2012). Briefly, 100 mg of root tissue was ground in 5 mL of 100 mM phosphate buffer (pH 7.0). The homogenate was then centrifuged for 10 min before separating the supernatant in Eppendorf tubes. For CAT analysis, the reaction mixture (2 mL) contained 100 mM potassium phosphate buffer (pH 7.0), 400 μl of 6% H_2_O_2_, and 100 μl root extract. After adding the root extract, the decrease in absorbance was recorded at 240 nm (extinction coefficient of 0.036 mM^-1^ cm^-1^) using a UV spectrophotometer at 30 s intervals up to 1 min. The activity of CAT is expressed as μmol of H_2_O_2_ oxidized min^-1^ (mg protein)^-1^. In the case of POD, the reaction mixture (2 mL) contained 100 mM potassium phosphate buffer (pH 6.5), 1 ml of 0.05 M pyrogallol solution, 400 μl of 200 mM H_2_O_2_, and 100 μl root extract. Similarly, the changes in absorbance were read at 430 nm (extinction coefficient 12 mM^-1^cm^-1^) in a spectrophotometer from 30 s up to 1.5 min. The specific activity of the enzyme is expressed as μmol pyrogallol oxidized min^-1^ (mg protein)^-1^. Further, SOD assay mixture contained 50 mM sodium carbonate/bicarbonate buffer (pH 9.8), 0.1 mM EDTA, 0.6 mM epinephrine and enzyme (Sun and Zigman, 1978). Once epinephrine is added, adrenochrome formation for 4 min was then read at 475 nm in a UV-Vis spectrophotometer. For GR activity, 100 μl of root extract was added the assay mixture contained 1 mL of 0.2 M phosphate buffer (pH 7.0) with 1 mM EDTA, 0.75 ml distilled water, 0.1 mL of 20 mM oxidized glutathione (GSSG), and 0.1 mL of 2 mM NADPH. Oxidation of NADPH by GR was then monitored at 340 nm. The GR activity was then calculated using the extinction coefficient of 6.12 mM^-1^ cm^-1^ (Halliwell and Foyer, 1978). For H_2_O_2_ determination, tissues were homogenized in 0.1% trichloroacetic acid (TCA) at 4 °C. The samples were then centrifuged 10,000 × *g* for 15 min to separate supernatant. The samples were then mixed with phosphate buffer (10 mM, pH 7.0) and potassium iodide (1 M) for 1 h. The absorbance of the solution mixture was recorded at 390 nm (Alexieva et al., 2001).

### Analysis of Plant Metabolites by HPLC (High-Performance Liquid Chromatography)

Plant metabolites were analyzed in roots by HPLC (Binary Gradient HPLC System, Waters Corporation, Milford, MA, USA) with Empower2^TM^ software as previously described with some modifications (Kabir et al., 2015; Kabir, 2016). Briefly, samples were ground in mortar and pestle using deionized water and centrifuged at 1500 × *g* for 10 min before separating the supernatant in Eppendorf tubes. The HPLC systems comprised a Waters 515 HPLC pump and Waters In-line degasser AF. For compound separation, a C18 reverse phase-HPLC column (particle size: 5 μm, pore size: 300 A, pH Range: 1.5–10, Dimension: 250 mm × 10 mm) was attached. In mobile-phase, buffer A (water and 0.1% TFA) and buffer B (80% acetonitrile and 0.1% TFA) were used at the gradient of: 1–24 min 100% A, 25–34 min 100% B, and 35–40 min 100% A. Standards and samples were diluted (100×) and subsequently filtered using 0.22 μm Minisart Syringe Filters (Sartorius Stedim Biotech, Germany) before injection. The retention time of each metabolite was then detected with a Waters 2489 dual absorbance detector (Waters Corporation, Milford, MA, USA) at 280 and 360 nm. Further, PC was identified by the retention time observed for both PC standard solution and the extract of hyperaccumulating plant *Noccaea caerulescens* (formerly known as *Thlaspi arvense*) to check the consistency of the peak (Supplementary Table S2). Since the HPLC detects both GSH and PC, GSH-equivalents of PC were used for PC quantification (Lindberg et al., 2007).

### RNA Isolation and Real-Time PCR Analysis

Expression analysis of *Actin, TaSAMS, TaDMAS1, TaYSL15, TaPCS1. and TaMT1* was performed by quantitative qRT-PCR (reverse transcription PCR). Briefly, tissues (50–100 mg) were ground with a mortar and pestle to a fine powder in liquid nitrogen. Afterward, total RNA was isolated as instructed by SV Total RNA Isolation System (cat. no. Z3100), Promega Corporation, USA. The integrity of isolated RNA was then checked by denaturing agarose gel electrophoresis and quantified by NanoDrop 2000 UV-Vis Spectrophotometer. The first-strand cDNA was then synthesized by using GoScript^TM^ Reverse Transcription System (Cat no. A5001), Promega Corporation, USA. Before real-time analysis, the cDNA samples were treated with RNAase for removing RNA contamination. Real-time PCR was performed in triplicate using GoTaq^®^qPCR Master Mix (Promega USA) and gene-specific primers (Supplementary Table S1) in an Eco^TM^ real-time PCR system (Illumina, USA). Expression data was normalized with *Actin* as an internal control (Eco Software v4.0.7.0). The real-time PCR program used was as follows: 3 min at 95°C, 40 cycles of 30 s at 94°C, 15 s at 58°C, and 30 s at 72°C.

### Reciprocal Grafting of Contrasting Genotypes

Reciprocal grafting was performed on newly germinated plants as previously described (Kabir et al., 2013; Begum et al., 2016) before transferring to the solution culture (**Supplementary Figure S2**). Briefly, the emerging shoot of BR 27 and BR 26 was diagonally cut (45° from the horizontal) 0.5 cm above the germinated seed. Scion (the portion removed) separated from each genotype was grafted onto each genotype’s rootstock in four combinations: BR 27 rootstock + BR 27 scion (type 1), BR 26 rootstock + BR 26 scion (type 2), BR 27 rootstock + BR 26 scion (type 3), and BR 26 rootstock + BR 27 scion (type 4). Each graft was detained together by thin silicon tube positioned over the graft. Any grafted plants where the graft had separated due to growth or bending were not taken for analysis.

Grafted plants were further tested through RAPD (random amplified polymorphic DNA) analysis in the shoot to confirm the source of scion used in each type of grafts (**Supplementary Figure S3**). Briefly, around 100 mg of shoot tissues was ground to a fine powder in liquid nitrogen using a mortar and pestle. The genomic DNA was extracted as instructed by Wizard^®^Genomic DNA Purification Kit (cat. no. A1120), Promega Corporation, USA. Isolated DNA was then amplified with RAPD primer in a MultiGene^TM^ OptiMax Thermal Cycler, Labnet International Inc. The PCR program used was as follows: 2 min at 94°C, 40 cycles of 30 s at 94°C, 30 s at 27°C, 1 min at 72°C, and 10 min at 72°C. The PCR products from each grafted plants (types 1–4) were run in 0.8% agarose gel electrophoresis and visualized in Gel documentation system (AlphaImager Mini System, ProteinSimple, San Jose, CA, USA).

### Application of Auxin Inhibitor

Once seedlings were transferred to the solution culture, 2.5 μM TIBA (polar auxin transport inhibitor) mixed with lanolin was applied to stem with reapplication in every 2 days (Yin et al., 2011). Lanolin was also used to the plants not treated with an inhibitor to nullify the effect (if any) of lanolin itself.

### Analysis of Plant Hormones by LC-MS (Liquid Chromatography–Mass Spectrometry)

Indole-3-acetic acid (AAA) and Indole-3-butyric acid (IBA) were analyzed by LCMS-2020 equipped with Prominence HPLC system (Shimadzu, Kyoto, Japan) as previously described with some modifications based on a standard curve (Begum et al., 2016; Kabir et al., 2016). Briefly, a mobile-phase column (EZ:faast AAA-MS column 250 × 2.0 mm) was eluted (0.25 mL/min) with (A) 10 mM ammonium formate in water, and (B) 10 mM ammonium formate in methanol. The temperature of column and autosampler tray was kept constant at 35 and 10°C, respectively. A gradient starting at 68–83% (B) over 13 min, and then ramping to 68% by 13.01 min was used for analyses The column was then allowed to re-equilibrate at 68% (B) for 4 min. The MS was operated with nebulizer gas flow of 1.5 L/min, drying gas flow of 15 L/min, ESI voltage of 1.8 kV, temperature of 365°C. Samples were ionized by positive ion electrospray ionization mode under the following source conditions: Nebulizing gas flow 1.5 l/min, turbo gas flow of 80 L/min; detector voltage was fixed as tuning, gas temperature of 250°C, and heat block temperature 200°C. Mass spectra were obtained at selected ion monitoring (SIM) mode. Peak areas for all components were automatically integrated by using Shimadzu LC-MS lab solution software.

### Statistical Analysis

All experiments were performed in a completely randomized block design. Each sample had at least three independent biological replications. Data for inhibitor studies were analyzed by one-way analysis of variance followed by Duncan’s Multiple Range Test (DMRT) at *P* ≤ 0.05. In other experiments, statistical significance between control and treatment was analyzed by *t*-test at *P* ≤ 0.05. All the statistical analyses were performed using IBM SPSS Statistics 20 Software (SPSS Inc., Chicago, IL, USA). Further, graphical presentations were prepared by GraphPad Prism 6 software.

## Results

### Morpho-Physiological Features

Morphological growth parameters, such as root length, root dry weight, shoot height, and shoot dry weight showed significant genotypic variations in response to higher Fe concentration. These parameters did not show major changes in BR 27 in response to higher Fe concentration compared with control plants (**Table 1**). However, these growth features significantly decreased in BR 26 due to higher Fe concentration in comparison with control plants. Further, total chlorophyll concentration (a and b) showed no significant changes in response to higher Fe concentration compared with controls in both genotypes (**Table 1**).

**Table 1:** Morpho-physiological growth parameters in BR 27 and BR 26 grown under control and excess Fe (15 mM).

Parameters	BR 27		BR 26	
		
	Control	Fe (15 mM)	Control	Fe (15 mM)
Root length (cm)	10.1 ± 0.25a	8.6 ± 1.00a	8.8 ± 1.41a	5.9 ± 0.55b
Root dry weight (g)	0.033 ± 0.004a	0.030 ± 0.004a	0.029 ± 0.008a	0.011 ± 0.001b
Shoot height (cm)	11.5 ± 0.20a	9.9 ± 0.83a	11.4 ± 0.26a	8.3 ± 0.55b
Shoot dry weight (g)	0.073 ± 0.003a	0.062 ± 0.007a	0.094 ± 0.011a	0.044 ± 0.005b
Chlorophyll (a + b) in leaves (mg/g FW)	66.4 ± 23.4a	96.9 ± 30.6a	71.5 ± 5.1a	81.8 ± 25.0a


*Values are the means of three independent replicates with standard deviations. Different letters indicate significant differences between means ± SD of treatments (*n* = 3), comparisons were done for control and Fe (15 mM) conditions.*

### Fe Concentration and PS Release

**FIGURE 1:**
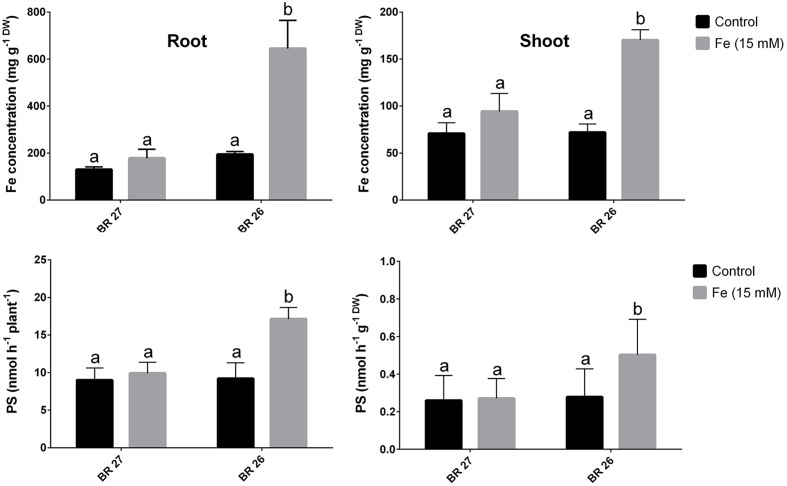
**Fe concentrations and phytosiderophores (PS) release in BR 27 and BR 26 grown under control and excess Fe (15 mM) in hydroponic conditions.** Different letters indicate significant differences between means of treatments (*n* = 3).

Fe concentration showed no significant changes in roots or shoots of BR 27 plants when plants were grown under higher Fe concentration compared with the plants grown under control conditions (**Figure 1**). In contrast, BR 26 plants showed a significant increase in Fe concentrations in both roots and shoots in response to higher Fe concentration compared with controls. Again, PS release (per plant or gram root weight) was not significantly changed in roots of BR 27 under excess Fe compared with controls. However, higher Fe concentration caused a significant increase in PS release in roots of BR 26 compared with non-treated controls (**Figure 1**).

### Changes in Biochemical Characteristics

**FIGURE 2:**
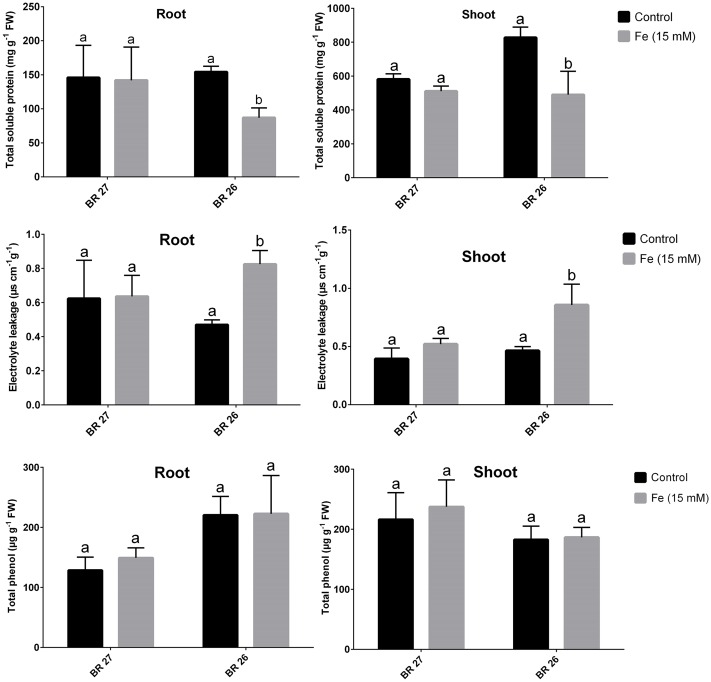
**Total soluble protein, electrolyte leakage, total phenol content in BR 27 and BR 26 grown under control and excess Fe (15 mM).** Different letters indicate significant differences between means of treatments (*n* = 3).

Total soluble protein and EL were not changed in both roots and shoots in response to higher Fe concentration in BR 27; while these parameters showed significant decrease and increase, respectively, in both tissues in BR 26 compared with the control plants (**Figure 2**). However, total phenol content showed no significant changes in either roots or shoots of both genotypes between controls and excess Fe (**Figure 2**).

### Relative Expression of Candidate Genes in Contrasting Genotypes

**FIGURE 3:**
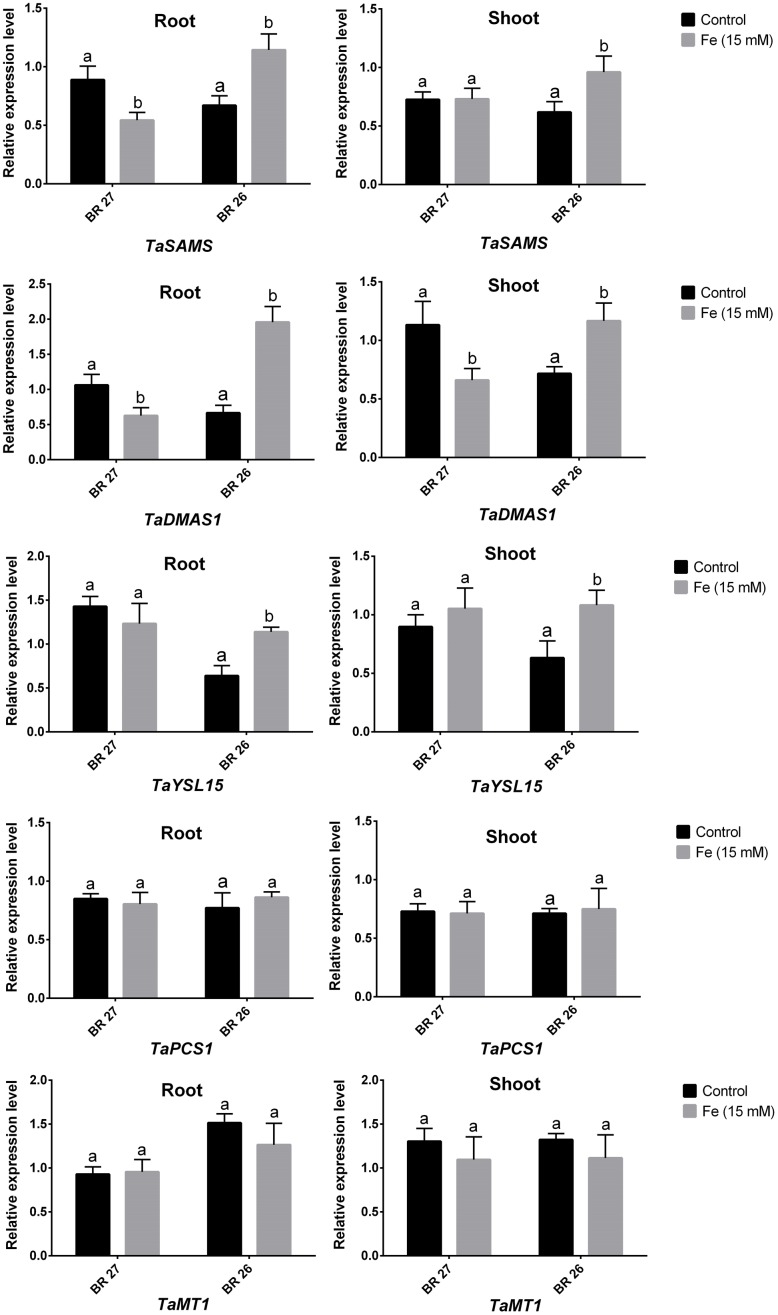
**Expression analysis of *TaSAMS, TaDMAS1, TaYSL15, TaPCS1. and TaMT1* transcripts in roots and shoots grown under control and excess Fe.** Different letters indicate significant differences between means of treatments (*n* = 3).

Real-time PCR analysis showed that three genes (*TaSAMS, TaDMAS1*, and *TaYSL15*) related to Fe acquisition showed significant downregulation in response to higher Fe concentration in roots of BR 27 compared with controls (**Figure 3**). However, expression of these genes significantly increased in roots of BR 26 under higher Fe in comparison with control plants. In shoots, *TaSAMS* and *TaYSL15* genes showed no changes, but *TaDMAS1* significantly downregulated under higher Fe concentration compared with controls in BR 27. In contrast, *TaSAMS, TaDMAS1*, and *TaYSL15* transcript showed a significant increase in shoots of BR 26 following higher Fe treatment compared with control plants. Another two genes, *TaPCS1* and *TaMT1* showed no significant changes in expression in roots or shoots of BR 27 and BR 26 in response to excess Fe (**Figure 3**).

### Changes in Plant Metabolites in Roots

Glutathione, cysteine, and proline significantly increased in roots of BR 27; whereas these metabolites showed no significant changes in BR 26 in response to excess Fe compared with control plants (**Table 2**). Methionine and 2-DMA were not significantly changed in roots of BR 27 but showed a significant increase in BR 26 in response to higher Fe concentration compared with controls. Total PC showed no significant changes in roots of any of the genotypes in response to excess Fe (**Table 2**).

**Table 2:** Analysis of plant metabolites in roots in contrasting wheat genotypes grown under control and excess Fe (15 mM).

Metabolites	BR 27			BR 26		
		
	Control	Fe (15 mM)	Fold ratio	Control	Fe (15 mM)	Fold ratio
Glutathione	16.5 ± 2.4a	47.2 ± 7.1b	2.8-fold	20.2 ± 5.7a	13.8 ± 2.2a	NA
Methionine	17.6 ± 2.6a	22.7 ± 2.4a	NA	36.1 ± 4.3a	64.1 ± 13.9b	1.7-fold
Cysteine	1.9 ± 0.91a	40.5 ± 10.8b	21.0-fold	1.6 ± 1.0a	1.1 ± 0.81a	NA
Proline	47.2 ± 6.8a	73.9 ± 6.5b	1.5-fold	57.3 ± 20.2a	51.4 ± 9.1a	NA
52-deoxymugineic acid	10.6 ± 3.8a	13.4 ± 1.3a	NA	8.5 ± 2.4a	37.9 ± 11.3b	4.4-fold
Phytochelatin	13.3 ± 4.3a	9.6 ± 3.1a	NA	8.6 ± 2.4a	7.7 ± 2.0	NA


*Values are the means of three independent replicates with standard deviations. Different letters indicate significant differences between means ± SD of treatments (*n* = 3), comparisons were done for control and Fe (15 mM) conditions.*

### Changes in Antioxidant Enzymes and H_2_O_2_ Content in Roots

CAT, POD, and GR activities significantly increased in roots of BR 27 in response to higher Fe concentration compared with controls. In contrast, excess Fe caused a significant decrease in CAT activity, but POD and GR were unchanged in roots of BR 26 compared with controls (**Table 3**). In addition, SOD activity showed no significant changes in any of the genotypes between control and excess Fe. Further, H_2_O_2_ content showed a significant decrease and increase in roots of BR 27 and BR 26, respectively, following higher Fe concentration compared with control plants (**Table 3**).

**Table 3:** Antioxidant enzymes and H_2_O_2_ in roots of contrasting genotypes grown under control and excess Fe (15 mM).

Metabolites	BR 27			BR 26		
		
	Control	Fe (15 mM)	Fold ratio	Control	Fe (15 mM)	Fold ratio
Catalase (CAT) min^-1^ [(mg protein)^-1^]	0.42 ± 0.36a	1.61 ± 0.12b	3.8-fold	1.34 ± 0.16a	0.51 ± 0.08b	2.6-fold
Peroxidase (POD) min^-1^ [(mg protein)^-1^]	0.57 ± 0.43a	3.52 ± 0.52b	6.1-fold	0.56 ± 0.14a	0.63 ± 0.08a	NA
Superoxide dismutase (SOD) [U.mg^-1^ protein]	0.10 ± 0.03a	0.13 ± 0.04a	NA	0.09 ± 0.01a	0.12 ± 0.03a	NA
Glutathione reductase (GR) [nmol.NADH.min^-1^ mg protein^-1^]	0.06 ± 0.01a	0.13 ± 0.03b	2.0-fold	0.07 ± 0.01a	0.11 ± 0.09a	NA
hydrogen peroxide (H_2_O_2_) (μmol g^-1^ FW)	0.37 ± 0.03a	0.09 ± 0.05b	3.8-fold	0.35 ± 0.04a	0.72 ± 0.13a	2.0-fold


*Values are the means of three independent replicates with standard deviations. Different letters indicate significant differences between means ± SD of treatments (*n* = 3), comparisons were done for control and Fe (15 mM) conditions.*

### Grafting Experiments

**FIGURE 4:**
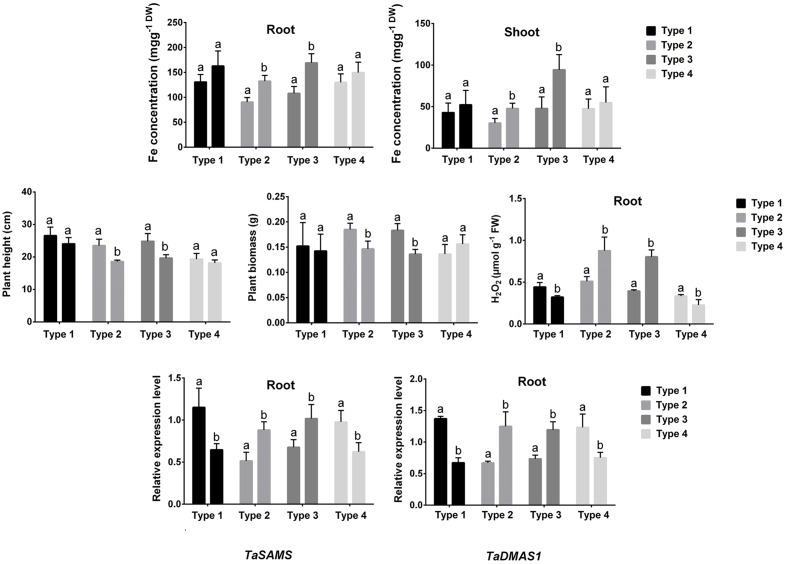
**Fe concentration, plant height, plant biomass, root H_2_O_2_ concentration, and expression of *TaSAMS* and *TaDMAS1* in different combination of grafted plants (type 1: BR 27 rootstock + BR 27 scion, type 2: BR 26 rootstock + BR 26 scion, type 3: BR 27 rootstock + BR 26 scion, type 4: BR 26 rootstock + BR 27 scion).** Different letters indicate significant differences between means ± SD of treatments (*n* = 3)

Fe concentrations and PS release showed no significant changes in roots of types 1 and 4; while these parameters significantly increased in types 2 and 4 grafts in response to higher Fe compared with the plants grown under control conditions (**Figure 4**). In addition, plant height and plant biomass were not significantly reduced in types 1 and 4; whereas types 2 and 4 grafts showed a significant decrease in these growth features following higher Fe compared with controls. Further, root H_2_O_2_ content showed a significant reduction in types 1 and 2 but significantly increased in types 3 and 4 subjected to higher Fe concentration compared with the control plants (**Figure 4**). Also, the expression of *TaSAMS* and *TaDMAS1* showed a significant decrease in types 1 and 2; whereas these genes were significantly upregulated in types 3 and 4 in response to excess Fe compared with controls (**Figure 4**).

### Effect of Auxin Inhibitor

**FIGURE 5:**
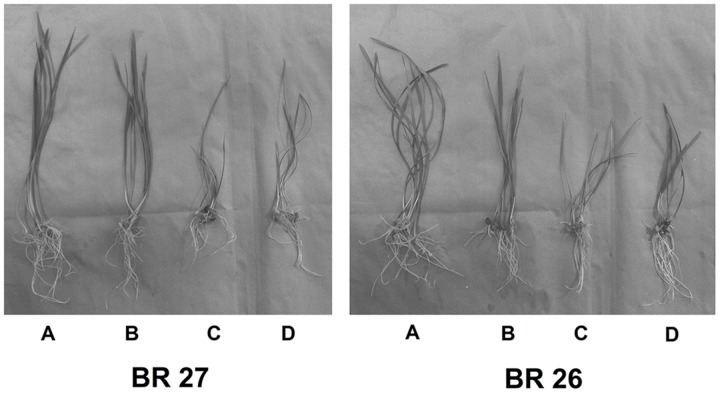
**Phenotype of plants grown under control, excess Fe, auxin inhibitor in hydroponic conditions.** **(A)** Control, **(B)** Fe (15 mM), **(C)** Fe (15 mM) + auxin inhibitor, **(D)** Control + auxin inhibitor.

**FIGURE 6:**
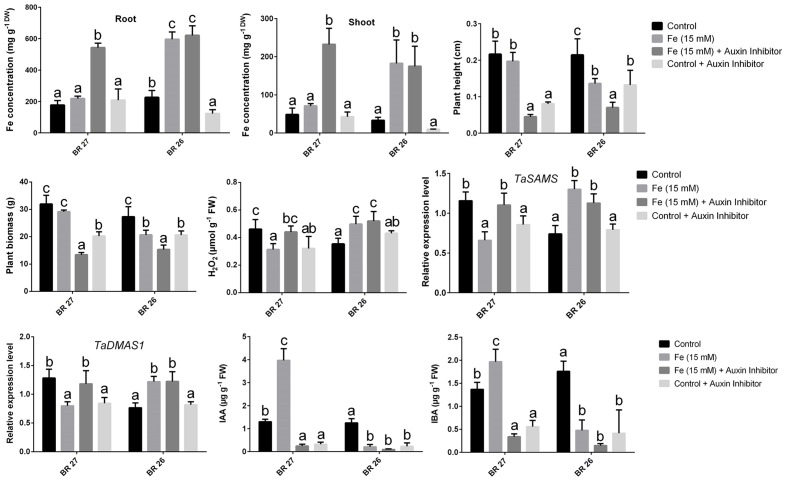
**Fe concentration, plant height, plant biomass, H_2_O_2_ concentration, gene expression, IAA, and IBA concentration in roots of BR 27 and BR 26 grown under different growth conditions.** Different letters indicate significant differences between means ± SD of treatments (*n* = 3) at a *P* < 0.05 significance level calculated by ANOVA followed by Duncan’s Multiple Range Test (DMRT).

A number of key parameters was studied in both BR 27 and BR 26 subjected to different growth conditions of Fe and auxin inhibitor. Although excess Fe did not show a significant increase in root and shoot Fe concentrations in BR 27, application of auxin inhibitor under excess Fe showed a significant increase in Fe concentration in roots and shoots compared with the plants grown solely under higher Fe concentration (**Figures 5** and **6**). In addition, plant height and plant biomass significantly decreased when both BR 27 and BR 26 plants were treated with auxin inhibitor under excess Fe compared with the plants solely stressed with excess Fe (**Figures 5** and **6**). Furthermore, application of auxin inhibitor under higher Fe concentration showed a significant increase in root H_2_O_2_ level compared with the plants grown under excess Fe in BR 27. Further, application of auxin inhibitor with excess Fe in BR 26 plants showed significant upregulation of *TaSAMS* and *TaDMAS1* genes compared with the plants grown solely under higher Fe (**Figure 6**). LC-MS analysis further demonstrated that IAA and IBA concentration significantly increased in response to excess Fe compared with controls in roots of BR 27, while these phytohormones were decreased in BR 26 plants (**Figure 6**). However, application of auxin inhibitor with or without higher Fe showed a significant reduction in IAA and IBA concentrations in roots of BR 27 compared with the plants treated with higher Fe. BR 26 plants treated with or without auxin inhibitor under excess Fe showed similar IAA and IBA levels to that of plants grown with excess Fe (**Figure 6**).

## Discussion

The response of plants to excess Fe is a complicated process and genotype dependent. However, concrete evidence associated with Fe-toxicity tolerance was lacking in wheat. In the present study, higher Fe concentration severely decreased growth parameters of BR 26 but not in BR 27. However, the chlorophyll concentration was not affected due to excess Fe in any of the genotypes suggesting that wheat photosynthetic mechanisms are not vulnerable to the high level of Fe. We observed variations in total soluble protein and EL in contrasting wheat genotypes in response to higher Fe. Higher Fe concentration caused no hamper in protein content in either roots or shoots of BR 27 plants, while BR 26 plants showed a significant reduction in protein content. Maintenance of protein level in BR 27 further implies that this genotype does have the ability to withstand excess Fe from cellular damage. The continuation of protein level under higher Fe concentration is possibly due to the induction of stress proteins, which may comprise various antioxidant enzymes and metabolites (Lamhamdi et al., 2010). Baruah et al. (2001) also reported that rice plants tolerant to higher Fe showed consistent total soluble protein in shoots. EL, a common damage in plasma membrane due to metal toxicity, was significantly changed in BR 27 plants under excess Fe. It implies that BR 27 plants are not affected by excess Fe and can maintain membrane stability in both roots and shoots.

To validate the above morphological and biochemical variations in BR 27 and BR 26, we further analyzed the Fe concentration and PS release following higher Fe treatment. These results revealed distinct differences in Fe accumulation in both roots and shoots in response to higher Fe in BR 27 and BR 26 genotypes. Fe was not significantly changed in roots and shoots of BR 27, while BR 26 showed a significant increase in Fe concentration due to excess Fe in comparison with control plants. These results are consistent with the PS release (**Figure 1**) and 2-DMA accumulation (**Table 2**) in roots revealing no changes in BR 27 roots following higher Fe treatment. DMA is generally induced under Fe deficiency in graminaceous plants (Astolfi et al., 2003, 2006; Ciaffi et al., 2013). In wheat, PS (non-protein amino acids) release from roots is the principal mechanisms for Fe acquisition in wheat. The genotypic variations in tolerance to Fe deficiency in cereal species are correlated with the PS rate from roots (Marschner et al., 1986; Kawai et al., 1988). The method we adopted for PS analysis is similar to those of Valentinuzzi et al. (2015) where distilled water was found to be the most suitable as trap solution for collecting root exudates from plants grown in hydroponic conditions.

Further, genes (*TaSAMS, TaDMAS1*, and *TaYSL15*) related to PS synthesis and transport showed opposite expression pattern in response to higher Fe concentration in BR 27 and BR 26 genotypes. In support of the biochemical data on PS release and 2-DMA synthesis, expression of these PS-related genes showed significant downregulation in roots of BR 27, while BR 26 showed significant downregulation in response to excess Fe. S-adenosylmethionine synthetase (SAMS) is involved in the the synthesis of S-adenosylmethionine (SAM) and DMA biosynthesis pathway (Heby and Persson, 1990; Takizawa et al., 1996). SAMS genes are differentially expressed in different organs/parts of plants (Peleman et al., 1989; Dekeyser et al., 1990) and also upregulated in Strategy II species under Fe deficiency (Suzuki et al., 2006; Li et al., 2014).

Like all other genes involved in DMA biosynthetic pathway, DMAS genes are upregulated following Fe shortage in root tissue in Strategy II plants (Bashir and Nishizawa, 2006). Also, expression of genes involved in the synthesis of MA (*HvNAAT-A* and *HvDMAS1*) and in the Fe uptake (*HvTOM1, HvYS1, HvIRT1, HvNramp2, HvNramp5*, and *HvNarmp7*) in barley upon Fe deficiency (Astolfi et al., 2010, 2014). However, DMA is also detected in Fe-sufficient shoot in rice possibly involved in Fe translocation as Fe-DMA complex (Mori et al., 1991). However, Bashir et al. (2014) reported that *OsDMAS1* showed downregulation under higher Fe in rice. *YSL15*, a dominant Fe-DMA transporter in rice, is upregulated under Fe deficiency and is associated with the transport of Fe(III)-PS and Fe(II)-NA complex (Inoue et al., 2009; Lee et al., 2009). In shoots, the expression pattern of *TaSAMS, TaDMAS1*, and *TaYSL15* was found similar to that of roots in both genotypes except for *TaSAMS* that showed no significant changes in roots of BR 27 in response to excess Fe. This is consistent with what was observed in AAS analysis, where inhibition of Fe uptake and translocation was observed in BR 27 under excess Fe. However, our findings first characterize that DMA and its related genes are also induced due to excess Fe in wheat. Moreover, it is strongly regulated to direct less Fe accumulation in Fe-toxicity tolerant BR 27 cultivar. Ciaffi et al. (2013) demonstrated that PS release was induced under Fe deficiency but the required methionine to facilitate PS production was associated with increased sulfate uptake capacity in wheat roots. In agreement with this report, we found no increase of methionine in roots of BR 27 under excess Fe, suggesting that the regulation of PS release might be correlated with root methionine level in roots and genotype specific in wheat. We also investigated the total PC content and the expression of *TaPCS1* and *TaMT1* if tolerance to excess Fe is also linked with metal chelation and sequestration in wheat. Interestingly, total PC content, as well as the expression of *TaPCS1* and *TaMT1*, showed no significant changes in response to higher Fe in any of the genotypes. These suggest that sequestration of Fe is not involved in the Fe-toxicity tolerance in wheat plants. Taken together, our results confirm that regulation of Fe acquisition mediated by PS release contributes major parts in differential tolerance to excess Fe in wheat plants.

The breakthrough of this study was to locate the origin of Fe-toxicity signal through the reciprocal grafting BR 27 and BR 26 plants. PCR-RAPD diagnostic confirmed the source of scion used in reciprocally grafted plants. In grafting experiments, parameters found most interesting underlying differential tolerance to excess Fe in BR 27 and BR 26 genotypes were picked up for analysis. In this present study, BR 27 type tolerance to excess Fe, as evident by growth parameters and Fe concentration, were fully observed in grafts comprising BR 26 rootstock and BR 27 scion. In contrast, plants grafted with BR 27 rootstock and BR 27 scion showed BR 26 type sensitivity to higher Fe concentration. Further, H_2_O_2_ content along with the expression of *TaSAMS* and *TaDMAS1* showed exhibited a consistent decrease in grafts having BR 27 scion (types 1 and 4); while grafts combined with BR 27 rootstock and BR 27 scion showed a significant increase in these parameters due to higher Fe compared with the control plants. These findings clearly indicate that BR 27 shoots do contain signal driving the tolerance mechanisms in roots in response to higher Fe in BR 27 plants. In contrast, BR 26 shoots might not deliver tolerance message to roots or BR 26 roots are unable to “sense” such signal in response to higher Fe concentration. In a similar grafting studies on Zn hyperaccumulator (*Thlaspi caerulescens*) and Zn non-accumulator (*Thlaspi perfoliatum*) revealed that Zn is primarily governed by root processes but mechanisms underlying Zn toxicity tolerance are driven by shoot based processed (De Guimarães et al., 2009). The present study gives the first evidence of the reciprocal grafting in wheat associated with Fe-toxicity and can be used for studying metal-induced signaling in crop plants.

High-Performance Liquid Chromatography analysis revealed changes of the main metabolites associated with Fe-toxicity tolerance in contrasting wheat genotypes. These results imply that tolerance to excess Fe in BR 27 is associated with metabolites having antioxidant properties, such as glutathione, cysteine, and proline. Glutathione plays important roles in cellular defense against toxicants, is known to increase under metal stress (Sun et al., 2007; Anjum et al., 2014). Further, glutathione and cysteine eliminated MDA content and prevented the toxic effects of As in rice seedlings (Shri et al., 2010). In addition, cysteine may lead to undesirable loss of sulfur, which potentially compromises the stress coping mechanisms of the plants (Zagorchev et al., 2013). Apart from acting as an osmolyte, proline scavenges free radicals, stabilizes sub-cellular structures, and proteins under stress conditions (Sharma and Dubey, 2005). Thus, our results suggest that accumulation of elevated antioxidative metabolites helps BR 27 seedlings to adapt better to the higher Fe stress.

Comparative analysis of antioxidant enzymes showed distinct genotypes variations under excess Fe. CAT, POD, and GR showed a significant increase in response to excess Fe in roots of BR 27 plants. In contrast, BR 26 showed significant decrease but no changes in POD, SOD, and GR in response to excess Fe. Induction of antioxidant enzymes is common in plants to withstand heavy metal stress (Panda and Choudhury, 2005; Shri et al., 2009). CAT catalyzes several cell reactions and facilitates the conversion of H_2_O_2_ to water and molecular oxygen Increased CAT activity has been reported under Pb stress in Vicia faba (Wang et al., 2010) and Cd stress in rice (Shah et al., 2001). Further, the activity of GR, which catalyzes the NADPH-dependent reduction of oxidized glutathione into glutathione (Rendon et al., 1995), is induced under metal stresses in plants (Dixit et al., 2001; Laspina et al., 2005). It also maintains the optimum level of glutathione for scavenging of ROS by other enzymes in plant cells. In this study, the H_2_O_2_ content was significantly reduced in roots of BR 27 under excess Fe in comparison with control plants. This might be attributed to significant increase in POD activity, the enzyme responsible for degradation of lipid peroxides. In the light of these findings, our data confirms that ROS-scavenging antioxidant metabolites and enzymes play critical roles for removing the H_2_O_2_ and thus, contribute partial tolerance to excess Fe in BR 27 plants. However, the inefficiency of BR 26 to provide antioxidant defense under excess Fe provides, at least in part, to its sensitivity to Fe stress.

Phytohormones, either natural or synthetic, function as signals for activation or inhibition of growth and development in plants. Within auxin group, IBA and IAA are involved in cell division, leaf expansion, antioxidant activities, and stress tolerance (Tyburski et al., 2009; Iglesias et al., 2010; El-Gaied et al., 2013). In light of reciprocal grafting, the identification of the link between auxin and tolerance was further investigated subjected to auxin inhibitor. Our analysis revealed the significant increase of IAA and IBA in roots of BR 27 plants in response to excess Fe; while these hormones remarkably decreased due to auxin inhibitor in BR 26. Further, the application of auxin inhibitor caused a significant increase in tissue Fe and reduction in plant biomass in BR 27 plants. Similarly, the expression of *TaSAMS* and *TaDMAS1* significantly increased in roots of BR 27 due to auxin inhibitor under excess Fe compared to the plants grown under excess Fe. These indicate that inhibition of auxin signal from shoot to root severely affected the mechanisms conferring tolerance to excess Fe in BR 27. Also, auxin inhibitor showed an adverse effect on the H_2_O_2_ level in roots of BR 27 plants, suggesting that auxin might stimulate antioxidant activities in wheat plants in response to excess Fe. Similarly, auxin inhibitor reduced the tolerance to arsenic stress and increased the levels of H_2_O_2_ (Krishnamurthy and Rathinasabapathi, 2013) in *Arabidopsis*. In this present study, we observed a correlation of auxin inhibition with the reduced tolerance to excess in BR 27 genotype. Therefore, it suggests that auxin is the signal driving from shoot to root to trigger tolerance mechanisms to excess Fe in wheat plants. These findings may facilitate the modification of biosynthetic pathway of the particular hormone to generate stress-tolerant plants.

## Conclusion

Our results provide novel insight into the biochemical and molecular evidence underlying tolerance to excess Fe in wheat. Our study suggests that regulation of PS release through the downregulation of PS and 2-DMA related genes play an integral part for limiting Fe acquisition under excess Fe in wheat. Further, ROS damage caused by excess Fe was partially minimized in BR 27 by the increased activity of CAT, POD, and GR along with the elevated synthesis of glutathione, cysteine, and proline. Experiments on reciprocal grafting and auxin inhibitors further suggest a signaling role of auxin driving tolerance mechanisms in response to excess Fe in roots of BR 27. These findings will be influential in the development of strategies through transformation or auxin signaling to improve tolerance under excess Fe in Strategy II plants.

## Author Contributions

AK performed most of the experiments and prepared the draft manuscript. MK and MH conducted few laboratory experiments. SH, MA, and NP revised the draft manuscript.

## Conflict of Interest Statement

The authors declare that the research was conducted in the absence of any commercial or financial relationships that could be construed as a potential conflict of interest.

## Supplementary Material

**FIGURE S1 | Phenotype of contrasting wheat genotypes grown under different concentration of Fe to optimize stressed conditions.**

**FIGURE S2 | Reciprocal grafting of contrasting wheat genotypes.** **(A)** Rootstock, **(B)** Scion, **(C)** Glass tube, **(D)** Joining of rootstock and scion.

**FIGURE S3 | PCR diagnostic in shoot of different grafted plants through random amplified polymorphic DNA (RAPD) primer (ACGGTACCAG).**

## References

[B1] Alexieva V. Sergiev I. Mapelli S. Karanov E. (2001). The effect of drought and ultraviolet radiation on growth and stress markers in pea and wheat. *Plant Cell Environ.* 24 1337–1344. 10.1046/j.1365-3040.2001.00778.x

[B2] Anjum N. Aref I. M. Duarte A. C. Pereira E. Ahmad I. Igbal M. (2014). Glutathione and proline can coordinately make plants withstand the joint attack of metal(loid) and salinity. *Front. Plant Sci.* 5:662 10.3389/fpls.2014.00662 PMC4240066 25484889

[B3] Astolfi S. Cesco S. Zuchi S. Neumann G. Roemheld V. (2006). Sulfur starvation reduces phytosiderophores release by iron-deficient barley plants. *J. Plant Nutr.* 52 43–48.

[B4] Astolfi S. Ortolani M. R. Catarcione G. Paolacci A. R. Cesco S. Pinton R. (2014). Cadmium exposure affects iron acquisition in barley (*Hordeum vulgare*) seedlings. *Physiol. Plant.* 152 646–659. 10.1111/ppl.12207 24724721

[B5] Astolfi S. Zuchi S. Hubberten H. Pinton R. Hoefgen R. (2010). Supply of sulphur to S-deficient young barley seedlings restores their capability to cope with iron shortage. *J. Exp. Bot.* 61 799–806. 10.1093/jxb/erp346 20018904 PMC2814111

[B6] Astolfi S. Zuchi S. Passera C. Cesco S. (2003). Does the sulfur assimilation pathway play a role in the response to fe deficiency in maize (Zea mays L.) plants? *J. Plant Nutr.* 26 2111–2121. 10.1081/PLN-120024268

[B7] Audebert A. Sahrawat K. L. (2000). Mechanisms for iron toxicity tolerance in lowland rice. *J. Plant Nutr.* 23 1877–1885. 10.1080/01904160009382150

[B8] Aung T. (2006). *Physiological Mechanisms of Iron Toxicity Tolerance in Lowland Rice.* Ph.D. thesis, University of Bonn, Bonn.

[B9] Bacaicoa E. Mora V. Zamarreno A. M. Fuentes M. Casanova E. Garcia-Mina J. M. (2011). Auxin: a major player in the shoot-to-root regulation of root Fe-stress physiological responses to Fe deficiency in cucumber plants. *Plant Physiol. Biochem.* 49 545–556. 10.1016/j.plaphy.2011.02.018 21411331

[B10] Baruah K. K. Nath B. C. Gogoi N. (2001). “Physiological and biochemical traits of rice (Oryza sativa L.) genotypes associated with tolerance of iron toxicity,” in *Food Security and Sustainability of Agro-Ecosystems Through Basic and Applied Research. Book Chapter, Plant Nutrition-the Series Developments in Plant and Soil Sciences* Vol. 92 eds Horst W. J. Schenk M. K. Bürkert A. Claassen N. Flessa H. Frommer W. B. (Berlin: Springer), 476–477. 10.1007/s00122-015-2569-y

[B11] Bashir K. Hanada K. Shimizu M. Seki M. Nakanishi H. Nishizawa N. K. (2014). Transcriptomic analysis of rice in response to iron deficiency and excess. *Rice* 7:18 10.1186/s12284-014-0018-1 PMC4884027 26224551

[B12] Bashir K. Inoue H. Nagasaka S. Nishizawa N. K. (2006). Cloning and characterization of deoxymugineic acid synthase genes from graminaceous plants. *J. Biol. Chem.* 281 32395–32402. 16926158 10.1074/jbc.M604133200

[B13] Bashir K. Nishizawa N. K. (2006). Deoxymugineic acid synthase: a gene important for fe-acquisition and homeostasis. *Plant Signal. Behav.* 1 290–292. 10.4161/psb.1.6.3590 19704569 PMC2634242

[B14] Becker M. Asch F. (2005). Iron toxicity in rice-conditions and management concepts. *J. Plant Nutr. Soil Sci.* 168 558–573. 10.1002/jpln.200520504

[B15] Begum M. C. Islam M. S. Islam M. Amin R. Pavez M. S. Kabir A. H. (2016). Biochemical and molecular responses underlying differential arsenic tolerance in rice (*Oryza sativa* L.). *Plant Physiol. Biochem.* 104 266–277. 10.1016/j.plaphy.2016.03.034 27061371

[B16] Çelik H. Asik B. B. Gürel S. Katkat A. V. (2010). Potassium as an intensifying factor for iron chlorosis. *Int. J. Agric. Biol.* 12 359–364.

[B17] Ciaffi M. Paolacci A. R. Celletti S. Catarcione G. Kopriva S. Astolfi S. (2013). Transcriptional and physiological changes in the S assimilation pathway due to single or combined S and Fe deprivation in durum wheat (*Triticum durum* L.) *seedlings*. *J. Exp. Bot.* 64 1663–1675. 10.1093/jxb/ert027 23390290 PMC3617832

[B18] Cobbett C. S. (2000). Phytochelatins and their roles in heavy metal detoxification. *Plant Physiol.* 123 825–832. 10.1104/pp.123.3.825 10889232 PMC1539264

[B19] Connolly E. L. Guerinot M. L. (2002). Iron stress in plants. *Gen. Biol.* 3 1–6. 10.1186/gb-2002-3-8-reviews1024 PMC139400 12186653

[B20] Curie C. Panaviene Z. Loulergue C. Dellaporta S. L. Briat J. F. Walker E. L. (2001). Maize yellow stripe1 encodes a membrane protein directly involved in Fe-III uptake. *Nature* 409 346–349. 10.1038/35053080 11201743

[B21] Cuypers A. Smeets K. Ruytinx J. Opdenakker K. Keunen E. Remans T. (2011). The cellular redox state as a modulator in cadmium and copper responses in *Arabidopsis thaliana* seedlings. *J. Plant Physiol.* 168 309–316. 10.1016/j.jplph.2010.07.010 20828869

[B22] Dakora F. D. Phillips D. A. (2002). Root exudates as mediators of mineral acquisition in low-nutrient environments. *Plant Soil* 245 35–47. 10.1023/A:1020809400075

[B23] Dat J. F. Vandenabeele S. Vranova E. Van Montagu M. Inze D. Van Breusegem F. (2000). Dual action of the active oxygen species during plant stress responses. *Cell. Mol. Life Sci.* 57 779–795. 10.1007/s000180050041 10892343 PMC11147059

[B24] De Guimarães A. M. A. Gustin J. L. Salt D. E. (2009). Reciprocal grafting separates the roles of the root and shoot in zinc hyperaccumulation in *Thlaspi caerulescens*. *New Phytol.* 184 323–329. 10.1111/j.1469-8137.2009.02969.x 19656301 PMC2784906

[B25] De Tullio M. C. Jiang K. Feldman L. J. (2010). Redox regulation of root apical meristem organization: connecting root development to its environment. *Plant Physiol. Biochem.* 48 328–336. 10.1016/j.plaphy.2009.11.005 20031434

[B26] Dekeyser R. A. Claes B. De Rycke R. M. Habets M. E. Van Montagu M. C. (1990). Transient gene expression in intact and organized rice tissues. *Plant Cell* 2 591–602. 10.2307/3869123 12354966 PMC159914

[B27] Dixit V. Pandey V. Shyam R. (2001). Differential antioxidative responses to cadmium in roots and leaves of pea (*Pisum sativum* L. cv Azad). *J. Exp. Bot.* 52 1101–1109. 10.1093/jexbot/52.358.1101 11432926

[B28] Dobermann A. Fairhurst T. H. (2000). *Nutrient Disorders and Nutrient Management.* Manila: The International Rice Research Institute, 191.

[B29] El-Gaied L. F. Abu El-Heba G. A. El-Sherif N. A. (2013). Effect of growth hormones on some antioxidant parameters and gene expression in tomato. *GM Crops Food* 4 67–73. 10.4161/gmcr.24324 23549347

[B30] Emamverdian A. Ding Y. Mokhberdoran F. Xie Y. (2015). Heavy metal stress and some mechanisms of plant defense response. *Sci. World J.* 2015 756120 10.1155/2015/756120 PMC4321847 25688377

[B31] Fageria N. K. Carvalho G. D. Santos A. B. Ferreira E. P. B. Knupp A. M. (2011). Chemistry of lowland rice soils and nutrient availability. *Commun. Soil Sci. Plant Anal.* 42 1913–1933. 10.1080/00103624.2011.591467

[B32] Fang W. C. Wang J. W. Lin C. C. Kao C. H. (2001). Iron induction of lipid peroxidation and effects on antioxidative enzyme activities in rice leaves. *Plant Grow Regul.* 35 75–80. 10.1023/A:1013879019368

[B33] Finatto T. de Oliveira A. C. Chaparro C. da Maia L. C. Farias D. R. Woyann L. G. (2015). Abiotic stress and genome dynamics: specific genes and transposable elements response to iron excess in rice. *Rice* 8:13 10.1186/s12284-015-0045-6 PMC4385019 25844118

[B34] Freeman J. L. Persans M. W. Nieman K. Albrecht C. Peer W. Pickering I. J. (2004). Increased glutathione biosynthesis plays a role in nickel tolerance in *Thlaspi* nickel hyperaccumulators. *Plant Cell* 16 2176–2191. 10.1105/tpc.104.023036 15269333 PMC519206

[B35] Garrity D. P. Oldeman L. R. Morris R. A. Lanka D. (1986). “Rainfed lowland rice ecosystems: characterization and distribution,” in *Progress in Rainfed Lowland Rice* (Los Baños: The International Rice Research Institute), 3–23.

[B36] Goud P. B. Kachole M. S. (2012). Antioxidant enzyme changes in neem, pigeonpea and mulberry leaves in two stages of maturity. *Plant Signal. Behav.* 7 1258–1262. 10.4161/psb.21584 22895104 PMC3493408

[B37] Guy C. Haskell D. Neven L. Klein P. Smelser C. (1992). Hydration-state-responsive protein link cold and drought stress in spinach. *Planta* 188 265–270. 10.1007/BF00216823 24178264

[B38] Halliwell B. Foyer C. H. (1978). Properties and physiological function of a glutathion reductase purified from spinach leaves by affinity chromotography. *Planta* 139 9–17. 10.1007/BF00390803 24414099

[B39] Heby O. Persson L. (1990). Molecular genetics of polyamine synthesis in eukaryotic cells. *Trends Biochem. Sci.* 15 153–158. 10.1016/0968-0004(90)90216-X 2187296

[B40] Hoagland D. R. Arnon D. I. (1950). The water-culture method for growing plants without soil. *Calif. Agric. Exp. Stat. Cir.* 347 1–32.

[B41] Iglesias M. J. Terrile M. C. Bartoli C. G. D’Ippo’lito S. Casalongue C. A. (2010). Auxin signaling participates in the adaptative response against oxidative stress and salinity by interacting with redox metabolism in *Arabidopsis*. *Plant Mol. Biol.* 74 215–222. 10.1007/s11103-010-9667-7 20661628

[B42] Inoue H. Kobayashi T. Nozoye T. Takahashi M. Kakei Y. Suzuki K. (2009). Rice OsYSL15 is an iron-regulated iron(III)-deoxymugineic acid transporter expressed in the roots and is essential for iron uptake in early growth of the seedlings. *J. Biol. Chem.* 284 3470–3479. 10.1074/jbc.M806042200 19049971

[B43] Ishimaru Y. Masuda H. Bashir K. Inoue H. Tsukamoto T. Takahashi M. (2010). Rice metal-nicotianamine transporter, OsYSL2, is required for the long-distance transport of iron and manganese. *Plant J.* 62 379–390. 10.1111/j.1365-313X.2010.04158.x 20128878

[B44] Jin C. W. You G. Y. Zheng S. J. (2008). The iron deficiency-induced phenolics secretion plays multiple important roles in plant iron acquisition underground. *Plant Signal. Behav.* 3 60–61. 10.4161/psb.3.1.4902 19704773 PMC2633963

[B45] Kabir A. H. (2016). Biochemical and molecular changes in rice seedlings (*Oryza sativa* L.) to cope with chromium stress. *Plant Biol.* 18 710–719. 10.1111/plb.12436 26804776

[B46] Kabir A. H. Paltridge N. G. Rossener U. Stangoulis J. C. R. (2013). Mechanisms associated with Fe-deficiency tolerance and signaling in shoots of *Pisum sativum*. *Physiol. Plant.* 147 381–395. 10.1111/j.1399-3054.2012.01682.x 22913816

[B47] Kabir A. H. Rahman M. M. Haider S. A. Paul N. K. (2015). Mechanisms associated with differential tolerance to Fe deficiency in okra (*Abelmoschus esculentus* Moench). *Environ. Exp. Bot.* 112 16–26. 10.1016/j.envexpbot.2014.11.011

[B48] Kabir S. R. Rahman M. M. Tasnim S. Karim M. R. Khatun N. Hasan I. (2016). Purification and characterization of a novel chitinase from *Trichosanthes dioica* seed with antifungal activity. *Int. J. Biol. Macromol.* 84 62–68. 10.1016/j.ijbiomac.2015.12.006 26666429

[B49] Kawai S. Takagi S. Sato Y. (1988). Mugineic acid-family phytosiderophores in roots secretions of barley, corn and sorghum varieties. *J. Plant Nutr.* 11 633–642. 10.1080/01904168809363829

[B50] Kim S. A. Guerinot M. L. (2007). Mining iron: iron uptake and transport in plants. *FEBS Lett.* 581 2273–2280. 10.1016/j.febslet.2007.04.043 17485078

[B51] Kogure K. Yamauchi I. Tokumura A. (2004). Novel antioxidants isolated from plants of the genera *Ferula. Inula. Prangos* and *Rheum* collected in Uzbekistan. *Phytomedicine* 11 645–651. 10.1016/j.phymed.2003.09.004 15636179

[B52] Kohler A. Blaudez D. Chalot M. Martin F. (2004). Cloning and expression of multiple metallothioneins from hybrid poplar. *New Phytol.* 164 83–93. 10.1111/j.1469-8137.2004.01168.x 33873478

[B53] Krishnamurthy A. Rathinasabapathi B. (2013). Auxin and its transport play a role in plant tolerance to arsenite-induced oxidative stress in *Arabidopsis thaliana*. *Plant Cell Environ.* 36 1838–1849. 10.1111/pce.12093 23489261

[B54] Kumar A. Dwivedi S. Singh R. P. Chakrabarty D. Mallick S. Trivedi P. K. (2014). Evaluation of amino acid profile in contrasting arsenic accumulating rice genotypes under arsenic stress. *Biol. Plant.* 58 733–742. 10.1016/j.jhazmat.2012.06.049

[B55] Lamhamdi M. Bakrim A. Aarab A. Lafont R. Sayah F. (2010). A comparison of lead toxicity using physiological and enzymatic parameters on spinach (*Spinacia oleracea*) and wheat (*Triticum aestivum*) growth. *Moroccan J. Biol.* 6–7, 64–73.

[B56] Laspina N. V. Groppa M. D. Tomaro M. L. Benavides M. P. (2005). Nitric oxide protects sunflower leaves against Cd-induced oxidative stress. *Plant Sci.* 169 323–330. 10.1016/j.plantsci.2005.02.007

[B57] Lee S. Chiecko J. C. Kim S. A. Walker E. L. Lee Y. Guerinot M. L. (2009). Disruption of OsYSL15 leads to iron inefficiency in rice plants. *Plant Physiol.* 150 786–800. 10.1104/pp.109.135418 19376836 PMC2689993

[B58] Lequeux H. Hermans C. Lutts S. Verbruggen N. (2010). Response to copper excess in *Arabidopsis thaliana*: impact on the root system architecture, hormone distribution, lignin accumulation and mineral profile. *Plant Physiol. Biochem.* 48 673–682. 10.1016/j.plaphy.2010.05.005 20542443

[B59] Li G. Xu W. Kronzucker H. J. Shi W. (2015). Ethylene is critical to the maintenance of primary root growth and Fe homeostasis under Fe stress in *Arabidopsis*. *J. Exp. Bot.* 66 2041–2054. 10.1093/jxb/erv005 25711703 PMC4378635

[B60] Li Y. Wang N. Zhao F. Song X. Yin Z. Huang R. (2014). Changes in the transcriptomic profiles of maize roots in response to iron-deficiency stress. *Plant Mol. Biol.* 85 349–363. 10.1007/s11103-014-0189-6 24648157

[B61] Lichtenthaler H. K. Wellburn A. R. (1985). Determination of total carotenoids and chlorophylls a and b of leaf in different solvents. *Biochem. Soc. Trans.* 11 591–592. 10.1042/bst0110591

[B62] Lindberg S. Landberg T. Greger M. (2007). Cadmium uptake and induction of phytochelatins in wheat protoplasts. *Plant Physiol. Biochem.* 45 47–53. 10.1016/j.plaphy.2007.01.001 17303432

[B63] Liu K. Yue R. Yuan C. Liu J. Zhang L. Sun T. (2015). Auxin signaling is involved in iron deficiency-induced photocsynthetic inhibition and shoot growth defect in rice (*Oryza sativa* L.). *J. Plant Biol.* 58 391–401. 10.1007/s12374-015-0379-z

[B64] Lutts S. Kinet J. M. Bouharmont J. (1996). NaCl-induced senescence in leaves of rice (*Oriza sativa* L.) cultivar differing in salinity resistance. *Ann. Bot.* 78 389–398. 10.1006/anbo.1996.0134

[B65] Marschner H. Romheld V. Kissel M. (1986). Different strategies in higher plants in mobilization and uptake of iron. *J. Plant Nutr.* 9 695–713. 10.1080/01904168609363475

[B66] Memon A. R. Aktoprakligil D. Zdemur A. Vertii A. (2001). Heavy metal accumulation and detoxification mechanisms in plants. *Turk. J. Bot.* 25 111–121.

[B67] Mori S. Nishizawa N. K. Hayashi H. Chino M. Yoshimura E. Ishihara J. (1991). Why are young rice plants highly susceptible to iron deficiency? *Plant Soil* 130 143–156. 10.1007/BF00011869

[B68] Nakanishi H. Yamaguchi H. Sasakuma T. Nishizawa N. K. Mori S. (2000). Two dioxygenase genes, Ids3 and Ids2, from *Hordeum vulgare* are involved in the biosynthesis of mugineic acid family phytosiderophores. *Plant Mol. Biol.* 44 199–207. 10.1023/A:1006491521586 11117263

[B69] Panda S. K. Choudhury S. (2005). Chromium stress in plants. *Braz. J. Plant Physiol.* 17 95–102. 10.1590/S1677-04202005000100008

[B70] Peleman J. Saito K. Cottyn B. Engler G. Seurinck J. (1989). Structure and expression analyses of the S-adenosylmethionine synthetase gene family in *Arabidopsis thaliana*. *Gene* 84 359–369. 10.1016/0378-1119(89)90510-6 2482229

[B71] Pich A. Manteuffel R. Hillmer S. Scholz G. Schmidt W. (2001). Fe homeostasis in plant cell: does nicotianamine play multiple roles in the regulation of cytoplasmic Fe concentration? *Planta* 213 967–976. 10.1007/s004250100573 11722133

[B72] Reichman S. M. Parker D. R. (2006). Critical evaluation of three indirect assays for quantifying phytosiderophores released by the roots of Poaceae. *Eur. J. Soil Sci.* 28 844–853.

[B73] Rendon J. L. Pardo J. P. Mendoza-Hernandez G. Rojo-Dominguez A. Hernandez-Arana A. (1995). Denaturing behavior of glutathione reductase from cyanobacterium Spirulina maxima in guanidine hydrochloride. *Arch. Biochem. Biophys.* 318 264–270. 10.1006/abbi.1995.1229 7733653

[B74] Sahrawat K. L. (2004). Iron toxicity in wetland rice and the role of other nutrients. *J. Plant Nutr.* 27 1471–1504.

[B75] Sandalio L. M. Dalurzo H. C. Gomez M. Romero-Puertas M. C. del Rio L. A. (2001). Cadmium-induced changes in the growth and oxidative metabolism of pea plants. *J. Exp. Bot.* 52 2115–2126. 11604450 10.1093/jexbot/52.364.2115

[B76] Schaaf G. Ludewig U. Erenoglu B. E. Mori S. Kitahara T. von Wirén N. (2004). ZmYS1 functions as a proton-coupled symporter for phytosiderophoreand nicotianamine-chelated metals. *J. Biol. Chem.* 279 9091–9096. 10.1074/jbc.M311799200 14699112

[B77] Shah K. Kumar R. G. Verma S. Dubey R. S. (2001). Effect of cadmium on lipid peroxidation, superoxide anion generation and activities of antioxidant enzymes in growing rice seedlings. *Plant Sci.* 161 1135–1144. 10.1016/S0168-9452(01)00517-9

[B78] Sharma P. Dubey R. S. (2005). Lead toxicity in plants. *Braz. J. Plant Physiol.* 17 35–52. 10.1590/S1677-04202005000100004

[B79] Sharma S. S. Dietz K. (2006). The significance of amino acids and amino acid-derived molecules in plant responses and adaptation to heavy metal stress. *J. Exp. Bot.* 57 711–726. 10.1093/jxb/erj073 16473893

[B80] Shenker M. Fan T. W. Crowley D. E. (2001). Phytosiderophores influence on cadmium mobilization and uptake by wheat and barley plants. *J. Environ. Qual.* 30 2091–2098. 10.2134/jeq2001.2091 11790018

[B81] Shri M. Kumar S. Chakrabarty D. Trivedi P. K. Mallick S. Misra P. (2009). Effect of arsenic on growth, oxidative stress, and antioxidant system in rice seedlings. *Ecotoxicol. Environ. Saf.* 72 1102–1101. 10.1016/j.ecoenv.2008.09.022 19013643

[B82] Shri M. Tripathi P. Tripathi R. D. Trivedi P. K. Chakrabarty D. Tuli R. (2010). Transcriptomic and metabolomic shifts in rice roots in response to Cr (VI) stress. *BMC Genomics* 11:648 10.1186/1471-2164-11-648 PMC3224690 21092124

[B83] Song W. Y. Mendoza-Cozatl D. G. Lee Y. (2014). Phytochelatin-metal (loid) transport into vacuoles shows different substrate preferences in barley and arabidopsis. *Plant Cell Environ.* 37 1192–1201. 10.1111/pce.12227 24313707 PMC4123957

[B84] Sun M. Zigman S. (1978). An improved Spectrophotomeric assay for Superoxide dismutase based on epinephrine autoxidation. *Anal. Biochem.* 90 81–89. 10.1016/0003-2697(78)90010-6 727489

[B85] Sun Q. Ye Z. H. Wang X. R. Wong M. H. (2007). Cadmium hyperaccumulation leads to an increase of glutathione rather than phytochelatins in the cadmium hyperaccumulator. *J. Plant Physiol.* 164 1489–1498. 10.1016/j.jplph.2006.10.001 17207552

[B86] Suzuki M. Takahashi M. Tsukamoto T. Watanabe S. Matsuhashi S. Yazaki J. (2006). Biosynthesis and secretion of mugineic acid family phytosiderophores in zinc-deficient barley. *Plant J.* 48 85–97. 10.1111/j.1365-313X.2006.02853.x 16972867

[B87] Takizawa R. Nishizawa N. K. Nakanishi H. Mori S. (1996). Effect of iron deficiency on S-adenosylmethionine synthetase in barley roots. *J. Plant Nutr.* 19 1189–1200. 10.1080/01904169609365190

[B88] Tognetti V. B. Van Aken O. Morreel K. (2010). Perturbation in indole-3-butyric acid homeostasis by the UDP-glucosyltransferase UTG74E2 modulates *Arabidopsis* architecture and water stress tolerance. *Plant Cell* 22 2660–2679. 10.1105/tpc.109.071316 20798329 PMC2947170

[B89] Tyburski J. Dunajska K. Mazurek P. Piotrowska B. Tretyn A. (2009). Exogenous auxin regulates H2O2 metabolism in roots of tomato (*Lycopersicon esculentum* Mill.) seedlings affecting the expression and activity of CuZn-superoxide dismutase, catalase, and peroxidase. *Acta Physiol. Plant.* 31 249–260. 10.1007/s11738-008-0225-8

[B90] Valentinuzzi F. Cesco S. Tomasi N. Mimmo T. (2015). Influence of different trap solutions on the determination of root exudates in *Lupinus albus* L. *Biol. Fertil. Soils* 51 757–765. 10.1007/s00374-015-1015-2

[B91] Wang C. Tian Y. Wang X. Geng J. Jiang J. Yu H. (2010). Lead-contaminated soil induced oxidative stress, defense response and its indicative biomarkers in roots of *Vicia faba* seedlings. *Ecotoxicology* 19 1130–1139. 10.1007/s10646-010-0496-x 20431941

[B92] Wu L. Shhadi M. Y. Gregorio G. Mathhis E. Becker M. Frei M. (2014). Genetic and physiological analysis of tolerance to acute iron toxicity in rice. *Rice* 7:8 10.1186/s12284-014-0008-3 PMC4052628 24920973

[B93] Yin C. Wu Q. Zeng H. Xia K. Xu J. Li R. (2011). Endogenous auxin is required but supraoptimal for rapid growth of rice (*Oryza sativa* L.) seminal roots, and auxin inhibition of rice seminal root growth is not caused by ethylene. *J. Plant Grow. Regul.* 30 20–29. 10.1007/s00344-010-9162-z

[B94] Zagorchev L. Seal C. E. Kranner I. Odjakova M. (2013). A central role for thiols in plant tolerance to abiotic stress. *Int. J. Mol. Sci.* 14 7405–7432. 10.3390/ijms14047405 23549272 PMC3645693

