# Supplementary figures and images for: Regulation of Phytosiderophore Release and Antioxidant Defense in Roots Driven by Shoot-Based Auxin Signaling Confers Tolerance to Excess Iron in Wheat
Source: Front Plant Sci. 2016 Nov 10;7:1684. doi: 10.3389/fpls.2016.01684 (PMC5103167; doi:10.3389/fpls.2016.01684)

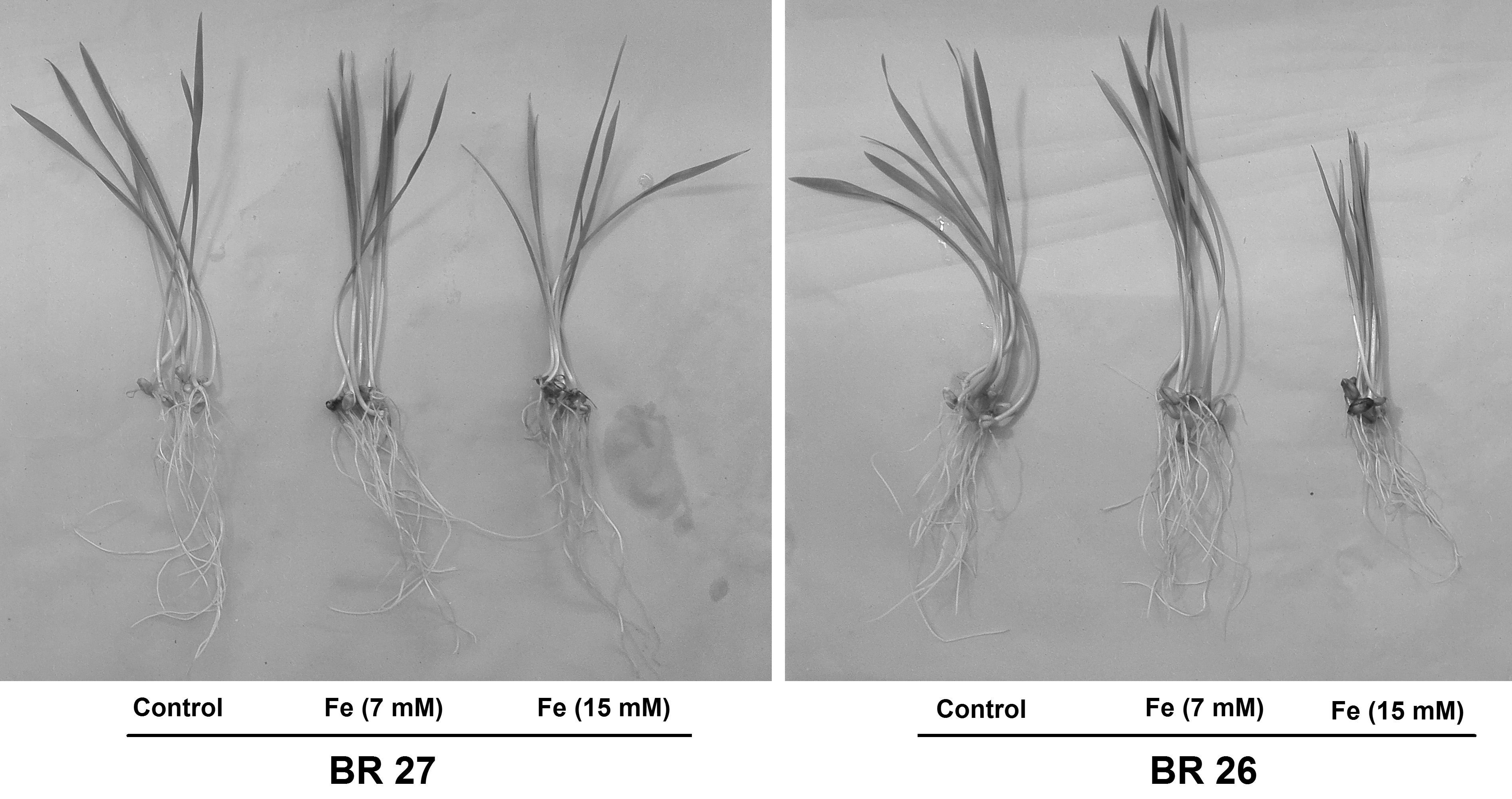

Supplement: Supplementary file 1 [file Image_1.JPEG]

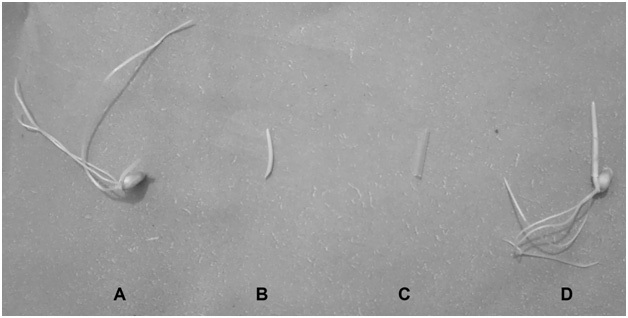

Supplement: Supplementary file 2 [file Image_2.JPEG]

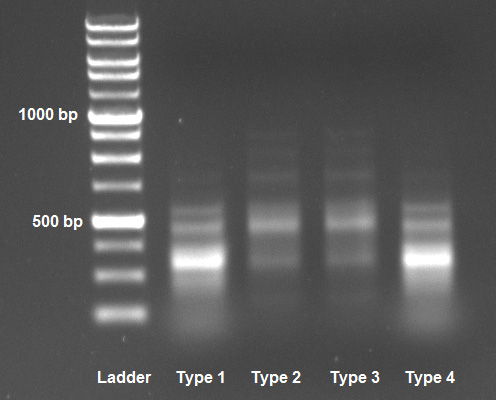

Supplement: Supplementary file 3 [file Image_3.JPEG]
