# Supplementary material for: Regulation of Phytosiderophore Release and Antioxidant Defense in Roots Driven by Shoot-Based Auxin Signaling Confers Tolerance to Excess Iron in Wheat
Source: Front Plant Sci. 2016 Nov 10;7:1684. doi: 10.3389/fpls.2016.01684 (PMC5103167; doi:10.3389/fpls.2016.01684)
Supplement: Supplementary file 4 [file Table_1.DOCX]

**Supplementary Table S1.** List of primers used for qPCR.

| Gene Name | Accession number | Primer sequences |
| --- | --- | --- |
| *Actin* | GQ339780.1 | Forward: TCTGTACGGCAATGTCGTGC  Reverse: ATCCCAGGGAACATGGTGG |
| *TaSAMS* | HM770448.1 | Forward: GCGTCCTCATCTACCAGAGC  Reverse: CTTGCCTTCCTTGACCAGAG |
| *TaDMAS1* | AB269908.1 | Forward: ACGTGGACCTGTACCTCGTC  Reverse: GGTTCACCTCCACCTGATTG |
| *TaYSL15* | AK334282.1  (highly similar to the rice *OsYSL15*) | Forward: GAGGATCCACACAACGTCAG  Reverse: CTGTAGGCGACAAC AAGCAA |
| *TaPCS1* | AF093752 | Forward: CAGACCACCATCCACGACTT  Reverse: ACAGCCTGTTCATTCCCTTT |
| *TaMT1* | L11879.1 | Forward: ACACCAAGGGCAGAGCATAG  Reverse: CACTCGTGTGATGGTGTGAG |
