# Supplementary material for: Regulation of Phytosiderophore Release and Antioxidant Defense in Roots Driven by Shoot-Based Auxin Signaling Confers Tolerance to Excess Iron in Wheat
Source: Front Plant Sci. 2016 Nov 10;7:1684. doi: 10.3389/fpls.2016.01684 (PMC5103167; doi:10.3389/fpls.2016.01684)
Supplement: Supplementary file 5 [file Table_2.DOCX]

**Supplementary Table S2.** Retention time of the metabolites analysed by HPLC systems.

| Name | Retention time (min) |
| --- | --- |
| Glutathione | 10.2 |
| Methionine | 28.3 |
| Cysteine | 12.4 |
| Proline | 35.9 |
| 2-deoxymugineic acid | 7.1 |
| Phytochelatin | 26.4 |
